# Semilocal Convergence Theorem for the Inverse-Free Jarratt Method under New Hölder Conditions

**DOI:** 10.1155/2015/346571

**Published:** 2015-03-25

**Authors:** Yueqing Zhao, Rongfei Lin, Zdenek Šmarda, Yasir Khan, Jinbiao Chen, Qingbiao Wu

**Affiliations:** ^1^Department of Mathematics, Taizhou University, Linhai, Zhejiang 317000, China; ^2^Department of Mathematics, Brno University of Technology, 616 00 Brno, Czech Republic; ^3^Department of Mathematics, Zhejiang University, Hangzhou, Zhejiang 310027, China

## Abstract

Under the new Hölder conditions, we consider the convergence analysis of the inverse-free Jarratt method in Banach space which is used to solve the nonlinear operator equation. We establish a new semilocal convergence theorem for the inverse-free Jarratt method and present an error estimate. Finally, three examples are provided to show the application of the theorem.

## 1. Introduction

We consider the following boundary value problem:

(1) $$\begin{array}{c} x^{\prime \prime }=-\lambda G\left(x\right),\\ x\left(a\right)=x_{a},\;\;x\left(b\right)=x_{b}. \end{array}$$

Those are equivalent to the following nonlinear integral equation (see [1, 2]):

(2) $$\begin{array}{c} x\left(s\right)=\alpha \left(s\right)+\lambda \overset{b}{\underset{a}{\int }}k\left(s,t\right)G\left(x\left(t\right)\right)dt, \end{array}$$

where *α*(*s*) = (1/(*b* − *a*))(*x*_*a*_(*b* − *s*) + *x*_*b*_(*s* − *a*)) and *G* : *Ω* ⊂ *C*[*a*, *b*] → *C*[*a*, *b*] is a twice Fréchet-differentiable operator. *C*[*a*, *b*] is the set of all continuous functions in [*a*, *b*]; *k*(*s*, *t*) is the Green function:

(3) $$\begin{array}{c} k\left(s,t\right)=\left\{\begin{array}{cc} \frac{\left(b-s\right)\left(t-a\right)}{b-a}, & t\leq s,\\ \frac{\left(s-a\right)\left(b-t\right)}{b-a}, & s\leq t. \end{array}\right. \end{array}$$

Instead of 2, we can try to solve a nonlinear operator equation *F*(*s*) = 0, where

(4) $$\begin{array}{c} F:\Omega \subset C\left[a,b\right]⟶C\left[a,b\right],\\ F\left(x\right)\left(s\right)=x\left(s\right)-\alpha \left(s\right)-\lambda \overset{b}{\underset{a}{\int }}k\left(s,t\right)G\left(x\left(t\right)\right)dt. \end{array}$$

Solving the nonlinear operator equation is an important issue in the engineering and technology field as these kinds of problems appear in many real-world applications. Economics [3], chemistry [4], and physics [5–8] are some of the examples of the scientific and engineering technology areas applied to solve these type of equations. In this study, we consider to establish a new semilocal convergence theorem of the Jarratt method in Banach space which is used to solve the nonlinear operator equation

(5) $$\begin{array}{c} F\left(x\right)=0, \end{array}$$

where *F* is defined on an open convex *Ω* of a Banach space *X* with values in a Banach space *Y*.

There are a lot of methods of finding a solution of equation *F*(*x*) = 0. Particularly iterative methods are often used to solve this problem (see [1, 2, 9, 10]). If we use the famous Newton method, we can proceed as

(6) $$\begin{array}{c} x_{n+1}=x_{n}-F^{\prime }{\left(x_{n}\right)}^{-1}F\left(x_{n}\right),\;\left(n\geq 0\right)\;\left(x_{0}\in \Omega \right). \end{array}$$

Under a reasonable hypothesis, Newton's method is the second-order convergence.

To improve the convergence order, many modified methods have been presented. The famous Halley's method and the supper-Halley method are the third-order convergence. References [11–22] give the convergence analysis for these methods. Now, we consider the following Jarratt method (see [23–25]):

(7) $$\begin{array}{c} y_{n}=x_{n}-F^{\prime }{\left(x_{n}\right)}^{-1}F\left(x_{n}\right),\\ H\left(x_{n},y_{n}\right)=\frac{3}{2}F^{\prime }{\left(x_{n}\right)}^{-1}\left[F^{\prime }\left(x_{n}+\frac{2}{3}\left(y_{n}-x_{n}\right)\right)-F^{\prime }\left(x_{n}\right)\right],\\ x_{n+1}=y_{n}-\frac{1}{2}H\left(x_{n},y_{n}\right)\left[I-H\left(x_{n},y_{n}\right)\right]\left(y_{n}-x_{n}\right). \end{array}$$

In this paper, we discuss the convergence of 7 for solving nonlinear operator equations in Banach spaces and establish a new semilocal convergence theorem under the following condition (see [20, 21]):

(8) $$\begin{array}{c} \left\|F^{\prime \prime \prime }\left(x\right)-F^{\prime \prime \prime }\left(y\right)\right\|\leq \omega \left(\left\|x-y\right\|\right), \end{array}$$

where *ω* : [0, +*∞*) → *R* is a nondecreasing continuous function. Finally, the corresponding error estimate is also given.

## 2. Main Results

In the section, we establish a new semilocal convergence theorem and present the error estimate. Denote *B*(*x*, *r*) = {*y* ∈ *X*∣‖*y* − *x*‖ < *r*} and $\bar{B(x,r)}=\{y\in X|\left\|y-x\right\|\leq r\}$. Suppose that *X* and *Y* are the Banach spaces, *Ω* is an open convex of the Banach space *X*, and *F* : *Ω* ⊂ *X* → *Y* has continuous Fréchet derivative of the third-order. *F*′(*x*_0_)^−1^ exists, for some *x*_0_ ∈ *Ω*, and *F* satisfies

(9)  A1y0−x0=F′x0−1F(x0)≤η; (A2)F′x0−1F′′(x)≤M, x∈Ω,  M≥0; (A3)F′x0−1F′′′(x)≤N, x∈Ω,  N≥0;A4F′x0−1F′′′x−F′′′y≤ωx−y,                 x,y∈Ω.

(A5) *ω*(*z*) is a nondecreasing continuous real function for *z* > 0 such that *ω*(0) ≥ 0, and there exists a positive real number *p* ∈ (0,1] such that *ω*(*tz*) ≤ *t* ^*p*^ *ω*(*z*) for *t* ∈ [0,1] and *z* ∈ [0, +*∞*).
(A6) Denote *A* = ∫_0_^1^∫_0_^1^ *t*(1 − *t*)(*st*)^*p*^ *ds* *dt* = (1/(*p* + 1)(*p* + 2)(*p* + 3)), *B* = (1/3)∫_0_^1^ ∫_0_^1^(2*st*/3)^*p*^ *t* *ds* *dt* = (2^*p*^/3^*p*+1^(*p* + 1)(*p* + 2)). Let *a*_0_ = *Mη*, *b*_0_ = *Nη* ^2^, *c*_0_ = *η* ^2^ *ω*(*η*), *a*_*n*+1_ = *a*_*n*_ *f* ^2^(*a*_*n*_)*g*(*a*_*n*_, *b*_*n*_, *c*_*n*_), *b*_*n*+1_ = *b*_*n*_ *f* ^3^(*a*_*n*_) *g* ^2^(*a*_*n*_, *b*_*n*_, *c*_*n*_), *c*_*n*+1_ = *f* ^3+*p*^(*a*_*n*_)*g* ^2+*p*^(*a*_*n*_, *b*_*n*_, *c*_*n*_), where
  (10) $$\begin{array}{c} f\left(x\right)=\frac{2}{2-2x-x^{2}-x^{3}},\\ g\left(x,y,z\right)=\frac{5x^{3}+2x^{4}+x^{5}}{8}+\frac{xy}{12}+\left(A+B\right)z. \end{array}$$

First, we get some lemmas.

**Lemma 1 .**

Suppose that *f*(*x*), *g*(*x*, *y*, *z*) are given by 10. Then

 ∀*x* ∈ (0, 1/2), *f*(*x*) is increasing and *f*(*x*) > 1;
 ∀*x* ∈ (0, 1/2), *y* > 0, *g*(*x*, *y*, *z*) is increasing;
 ∀*γ* ∈ (0,1), *x* ∈ (0, 1/2), *p* > 0, *f*(*γx*) < *f*(*x*) and *g*(*γx*, *γ* ^2^ *y*, *γ* ^2+*p*^ *z*)< *γ* ^2+*p*^ *g*(*x*, *y*, *z*).

**Lemma 2 .**

Suppose that *f*(*x*), *g*(*x*, *y*, *z*) are given by 10. If

(11) $$\begin{array}{c} a_{0}\in \left(0,\frac{1}{2}\right),\;f^{2}\left(a_{0}\right)g\left(a_{0},b_{0},c_{0}\right)<1, \end{array}$$

then

- the sequences {*a*_*n*_}, {*b*_*n*_}, {*c*_*n*_} are nonnegative and decreasing;
- (1 + (*a*_*n*_/2)(1 + *a*_*n*_))*a*_*n*_ < 1, ∀*n* ≥ 0.

**Proof**

(i) When *n* = 1,

(12) $$\begin{array}{c} 0\leq a_{1}=a_{0}f^{2}\left(a_{0}\right)g\left(a_{0},b_{0},c_{0}\right)\leq a_{0},\\ 0\leq b_{1}=b_{0}f^{3}\left(a_{0}\right)g^{2}\left(a_{0},b_{0},c_{0}\right)\leq b_{0},\\ 0\leq c_{1}=c_{0}f^{3+p}\left(a_{0}\right)g^{2+p}\left(a_{0},b_{0},c_{0}\right)\leq c_{0}. \end{array}$$

Suppose *a*_*j*_ ≤ *a*_*j*−1_, *b*_*j*_ ≤ *b*_*j*−1_ for *j* = 1,2,…, *n*. By Lemma 1, *f* and *g* are increasing; then

(13) an+1=anf2(an)g(an,bn,cn)≤anf2(a0)g(a0,b0,c0)≤an,bn+1=bnf3ang2an,bn,cn≤bnf3a0g2a0,b0,c0≤bn,cn+1=cnf3+p(an)g2+p(an,bn,cn)≤cnf2a0ga0,b0,c02+p≤cn.

(ii) By (i), {*a*_*n*_} is decreasing and *a*_0_ ∈ (0, 1/2). So, for all *n* ≥ 0,

(14) $$\begin{array}{c} \left(1+\frac{a_{n}}{2}\left(1+a_{n}\right)\right)a_{n}\leq \left(1+\frac{a_{0}}{2}\left(1+a_{0}\right)\right)a_{0}<1. \end{array}$$

This completes the proof of Lemma 2.

**Lemma 3 .**

Suppose that the conditions of Lemma 2 hold. Denote *γ* = *a*_1_/*a*_0_ = *f* ^2^(*a*_0_)*g*(*a*_0_, *b*_0_, *c*_0_) < 1. Then

- *a*_*n*_ ≤ *γ* ^(3 + *p*)^*n*−1^^ *a*_*n*−1_ ≤ *γ* ^(((3 + *p*)^*n*^ − 1)/(2 + *p*))^ *a*_0_, *b*_*n*_ ≤ (*γ* ^(3 + *p*)^*n*−1^^)^2^ *b*_*n*−1_ ≤ (*γ* ^(((3 + *p*)^*n*^ − 1)/(2 + *p*))^)^2^ *b*_0_, *c*_*n*_ ≤ (*γ* ^(3 + *p*)^*n*−1^^)^2+*p*^ *c*_*n*−1_ ≤ *γ* ^(3 + *p*)^*n*^−1^ *c*_0_ ∀ *n* ≥ 1;
- *f*(*a*_*n*_)*g*(*a*_*n*_, *b*_*n*_, *c*_*n*_) ≤ *γ* ^(3 + *p*)^*n*^−1^ *f*(*a*_0_)*g*(*a*_0_, *b*_0_, *c*_0_) = (*γ* ^(3 + *p*)^*n*^^/*f*(*a*_0_)), ∀*n* ≥ 1.

**Proof**

First, by induction, we prove that (i) holds. Because *a*_1_ = *γa*_0_ and *f*(*a*_0_) > 1, we have

(15) $$\begin{array}{c} b_{1}=b_{0}f^{3}\left(a_{0}\right)g^{2}\left(a_{0},b_{0},c_{0}\right)\leq \gamma^{2}b_{0},\\ c_{1}=c_{0}f^{3+p}\left(a_{0}\right)g^{2+p}\left(a_{0},b_{0},c_{0}\right)\leq \gamma^{2+p}c_{0}. \end{array}$$

Suppose that (i) holds for *n* ≥ 1. Then we get

(16) an+1=anf2angan,bn,cnan+1≤γ3+pn−1an−1f2γ3+pn−1an−1an+1<×gγ3+pn−1an−1,γ3+pn−12bn−1,γ3+pn−12+pcn−1an+1≤γ3+pn−1an−1f2an−1γ3+pn−12+pgan−1,bn−1,cn−1an+1=γ3+pnan−1f2an−1gan−1,bn−1,cn−1=γ3+pnan,an+1≤γ3+pnan≤γ3+pnγ3+pn−1an−1an+1≤⋯≤γ3+pnγ3+pn−1⋯γ3+p0a0an+1=γ3+pn+1−1/2+pa0,bn+1=bnf3(an)g2(an,bn,cn)≤bnan+1an2≤γ3+pn2bnbn+1≤⋯≤γ3+pn2γ3+pn−12⋯γ3+p02b0bn+1=γ3+pn+1−1/2+p2b0,cn+1=cnf3+p(an)g2+p(an,bn,cn)cn+1≤cnf2angan,bn,cn2+p=cnan+1an2+pcn+1≤γ3+pn2+pcn≤⋯≤γ3+pn+1−1c0

and from (ii) we get

(17) fangan,bn,cn ≤fγ3+pn−1/2+pa0  ×gγ3+pn−1/2+pa0,γ3+pn−1/2+p2b0,γ3n−1c0 ≤γ3+pn−1fa0ga0,b0,c0=γ3+pnfa0, n≥1.

This completes the proof of Lemma 3.

**Lemma 4 .**

Suppose that *X* and *Y* are Banach spaces, *Ω* is an open convex of the Banach space *X*, *F* : *Ω* ⊂ *X* → *Y* has continuous Fréchet derivative of the second-order, and the sequences {*x*_*n*_}, {*y*_*n*_} are generated by 7. Then, for all natural numbers *n* ≥ 0, the following formula holds:

(18) F(xn+1) =∫01F′′yn+txn+1−yn1−tdtxn+1−yn2  +∫01F′′xn+tyn−xn1−tdt    −12∫01F′′xn+23tyn−xndtyn−xn2  −12∫01xn+23tyn−xnF′′xn+tyn−xn      −F′′xn+23tyn−xndt     ×yn−xnHxn,ynyn−xn  +12∫01F′′xn+tyn−xndt     ×yn−xnHxn,ynHxn,ynyn−xn.

**Proof**

Consider

(19) Fyn =F(yn)−F(xn)−F′(xn)(yn−xn) =∫01F′′xn+tyn−xn1−tdtyn−xn2,F′(yn)(xn+1−yn) =−12[F′(yn)−F′(xn)]H(xn,yn)  ×[I−H(xn,yn)](yn−xn)  −12F′xnHxn,ynI−Hxn,ynyn−xn =−12∫01F′′(xn+t(yn−xn))dt    ×(yn−xn)H(xn,yn)(yn−xn)  +12∫01F′′(xn+t(yn−xn))dt    ×(yn−xn)H(xn,yn)H(xn,yn)(yn−xn)  −12∫01F′′xn+23tyn−xndtyn−xn2  +12∫01F′′xn+23tyn−xndt    ×yn−xnHxn,ynyn−xn,F(xn+1) =F(xn+1)−F(yn)−F′(yn)(xn+1−yn)  +F(yn)+F′(yn)(xn+1−yn) =∫01F′′(yn+t(xn+1−yn))(1−t)dtxn+1−yn2  +∫01F′′(xn+t(yn−xn))(1−t)dt    −12∫01F′′xn+23tyn−xndtyn−xn2  −12∫01xn+23tyn−xnF′′xn+tyn−xn      −F′′xn+23tyn−xndt      ×(yn−xn)H(xn,yn)(yn−xn)  +12∫01F′′xn+tyn−xndt    ×(yn−xn)H(xn,yn)H(xn,yn)(yn−xn).

This completes the proof of Lemma 4.

By (A1)–(A6), 10, and 11, if *a*_0_ < 1/2, then

(20) H(x0,y0)≤My0−x0=MF′x0−1F′(x0)y0−x0≤a0,x1−y0≤12H(x0,y0)I−H(x0,y0)y0−x0≤a02(1+a0)y0−x0,x1−x0≤x1−y0+y0−x0≤1+a021+a0y0−x0<Rη,

where *R* = [1 + (*a*_0_/2)(1 + *a*_0_)](1/(1 − *f*(*a*_0_)*g*(*a*_0_, *b*_0_, *c*_0_))); hence, *x*_1_, *y*_0_ ∈ *S*(*x*_0_, *Rη*). Consider

(21) F′x0−1F′x1−I ≤Mx1−x0≤1+a021+a0a0<1.

By Banach lemma, *F*′(*x*_1_)^−1^ exists, and

(22) $$\begin{array}{c} \left\|F^{\prime }{\left(x_{1}\right)}^{-1}F^{\prime }\left(x_{0}\right)\right\|\leq f\left(a_{0}\right)=f\left(a_{0}\right)\left\|F^{\prime }{\left(x_{0}\right)}^{-1}F^{\prime }\left(x_{0}\right)\right\|. \end{array}$$

By Lemma 4, we have

(23) ∫01F′′x0+23ty0−x0dtF′x0−1 ×∫01F′′x0+ty0−x01−tdt   −12F′x0−1 ×∫01F′′x0+23ty0−x0dt =∫01F′′x0+23ty0−x0−F′′x0dtF′x0−1∫01F′′−F′′x0x0+ty0−x0          −F′′x01−tdt   −12F′x0−1   ×∫01F′′x0+23ty0−x0−F′′x0dt =F′x0−1∬01F′′′−F′′′(x0)(x0+st(y0−x0))          −F′′′(x0)ds         ×t(1−t)dt(y0−x0)    −13F′x0−1    ×∬01F′′′x0+23sty0−x0       −F′′′(x0)F′′′x0+23sty0−x0ds t dt(y0−x0)F′x0−1∫01∫01 ≤(A+B)ω(η)y0−x0,F′x0−1F(x1) ≤M2x1−y02+N12a0y0−x03  +M2a02y0−x02+(A+B)ω(η)y0−x03,y1−x1 ≤F′x1−1F′(x0)F′x0−1F(x1) ≤f(a0)g(a0,b0,c0)y0−x0.

Hence,

(24) Hx1,y1≤MF′x1−1F′(x0)y1−x1     ≤Mf2(a0)g(a0,b0,c0)y0−x0=a1,NF′x1−1F′(x0)y1−x12 ≤Nf3a0g2a0,b0,c0y0−x02=b1,F′x1−1F′(x0)ωy1−x1y1−x12 ≤f3+pa0g2+pa0,b0,c0ωηy0−x02=c1.

Hence,

(25) x2−y1≤12a1(1+a1)y1−x1,x2−x1≤x2−y1+y1−x1≤1+12a11+a1y1−x1,x2−x0≤x2−x1+x1−x0≤1+a021+a0fa0ga0,b0,c0+1 ×y0−x0<Rη.

By

(26) F′x1−1F′(x2)−I≤MF′x1−1F′(x0)x2−x1 ≤a11+a121+a1<1,

hence *F*′(*x*_2_)^−1^ *F*′(*x*_0_) exists, and ‖*F*′(*x*_2_)^−1^ *F*′(*x*_0_)‖ ≤ *f*(*a*_1_)‖*F*′(*x*_1_)^−1^ *F*′(*x*_0_)‖. By induction, we can prove that the following Lemma 5 holds.

**Lemma 5 .**

Under the hypotheses of Lemma 2, the following items are true for all *n* ≥ 1:

- *F*′(*x*_*n*_)^−1^ *F*′(*x*_0_) exists and ‖*F*′(*x*_*n*_)^−1^ *F*′(*x*_0_)‖  ≤*f*(*a*_*n*−1_)‖*F*′(*x*_*n*−1_)^−1^ *F*′(*x*_0_)‖;
- ‖*y*_*n*_ − *x*_*n*_‖ ≤ *f*(*a*_*n*−1_)*g*(*a*_*n*−1_, *b*_*n*−1_, *c*_*n*−1_)‖*y*_*n*−1_ − *x*_*n*−1_‖;
- *H*(*x*_*n*_, *y*_*n*_) ≤ *M*‖*F*′(*x*_*n*_)^−1^ *F*′(*x*_0_)‖‖*y*_*n*_ − *x*_*n*_‖ ≤ *a*_*n*_;
- *N*‖*F*′(*x*_*n*_)^−1^ *F*′(*x*_0_)‖‖*y*_*n*_ − *x*_*n*_‖^2^ ≤ *b*_*n*_;
- ‖*F*′(*x*_*n*_)^−1^ *F*′(*x*_0_)‖*ω*(‖*y*_*n*_ − *x*_*n*_‖)‖*y*_*n*_ − *x*_*n*_‖^2^ ≤ *c*_*n*_;
- ‖*x*_*n*+1_ − *y*_*n*_‖ ≤ (*a*_*n*_/2)(1 + *a*_*n*_)‖*y*_*n*_ − *x*_*n*_‖;
- ‖*x*_*n*+1_ − *x*_*n*_‖ ≤ [1 + (*a*_*n*_/2)(1 + *a*_*n*_)]‖*y*_*n*_ − *x*_*n*_‖;
- ‖*x*_*n*+1_ − *x*_0_‖ ≤ *Rη*, where *R* = [1 + (*a*_0_/2)(1 + *a*_0_)] (1/(1 − *f*(*a*_0_)*g*(*a*_0_, *b*_0_, *c*_0_))).

**Theorem 6 .**

Let *X* and *Y* be two Banach spaces and *F* : *Ω* ⊂ *X* → *Y* has continuous Fréchet derivative of the third-order on a nonempty open convex *Ω*. One supposes that Γ_0_ = *F*′(*x*_0_)^−1^ ∈ *L*(*Y*, *X*) exists for some *x*_0_ ∈ *Ω* and conditions (A1)–(A6) and 11 hold. If $\bar{S(x_{0},R\eta )}\subset \Omega$, then the sequence {*x*_*n*_} generated by 7 is well defined and converges to a unique solution *x* ^*^ of 2 in *S*(*x*_0_, (2/*M*) − *Rη*)∩*Ω*. Furthermore, the following error estimate is obtained:

(27) x∗−xn ≤1+γ3+pn−1/2+pa021+γ3+pn−1/2+pa0  ×11−γ3+pnΔγ3+pn−1/2+pΔnη,

where *γ* = *f* ^2^(*a*_0_)*g*(*a*_0_, *b*_0_, *c*_0_) = *a*_1_/*a*_0_ and Δ = 1/*f*(*a*_0_), *R* = (1 + (*a*_0_/2)(1 + *a*_0_)) (1/(1 − *γ*Δ)).

**Proof**

Firstly, we prove that the sequence {*x*_*n*_} is a Cauchy one. From (II) and by Lemma 3, we have

(28) yn−xn≤fan−1gan−1,bn−1,cn−1yn−1−xn−1≤⋯≤∏i=0n−1faigai,bi,ciη≤∏i=0n−1γ3+piΔη=γ3+pn−1/2+pΔnη.

For *n* ≥ 0, *m* ≥ 1,

(29) xn+m−xn ≤xn+m−xn+m−1+xn+m−1−xn+m−2  +⋯+xn+1−xn ≤1+an21+an  ×yn+m−1−xn+m−1+⋯+yn+1−xn ≤1+an21+an  ×γ3+pn+m−1−1/2+pΔn+m−1    γ3+pn+m−1−1/2+pΔn+m−1+⋯+γ3+pn−1/2+pΔnη =1+an21+anγ3+pn−1/2+pΔnη  ×γ3+pn3+pm−1−1/2+pΔm−1+⋯+1.

By the Bernoulli inequality (1 + *x*)^*k*^ − 1 > *kx*, so (3 + *p*)^*k*^ − 1 > *k*(2 + *p*). Hence, we have

(30) xn+m−xn <1+an21+an11−γ3+pnΔγ3+pn−1/2+pΔnη.

Hence, {*x*_*n*_} is a Cauchy sequence and *x* ^*^ = lim⁡_*n*→*∞*_ *x*_*n*_. Obviously, *x*_*m*_ ∈ *B*(*x*_0_, *Rη*), for all *m* ≥ 1, as if *n* = 0 in 30; we obtain

(31) $$\begin{array}{c} \left\|x_{m}-x_{0}\right\|<\left(1+\frac{a_{0}}{2}\left(1+a_{0}\right)\right)\frac{1}{1-\gamma \Delta }\eta =R\eta . \end{array}$$

Following a similar procedure, we have *y*_*n*_ ∈ *B*(*x*_0_, *Rη*), for all *n* ≥ 0.

Now, let *n* → *∞* in 28. It follows that ‖*F*′(*x*_*n*_)^−1^ *F*(*x*_*n*_)‖ → 0. Besides ‖*F*(*x*_*n*_)‖ → 0, since ‖*F*(*x*_*n*_)‖ ≤ ‖*F*′(*x*_*n*_)‖‖*F*′(*x*_*n*_)^−1^ *F*(*x*_*n*_)‖ and {‖*F*′(*x*_*n*_)‖} is a bounded sequence, therefore *F*(*x* ^*^) = 0 by the continuity of *F* in $\bar{S(x_{0},R\eta )}$.

By letting *m* → *∞* in 30, we obtain error estimate 28.

To show uniqueness, let us assume that there exists a second solution *y* ^*^ of 2 in *S*(*x*_0_, (2/*M*) − *Rη*)∩*Ω*. Then

(32) ∫01F′x0−1F′x∗+ty∗−x∗−F′x0dt ≤M∫01x∗+t(y∗−x∗)−x0dt ≤M∫011−tx∗−x0+ty∗−x0dt <M2Rη+2M−Rη=1.

By Banach lemma, we can obtain that the inverse of the linear operator ∫_0_^1^ *F*′(*x* ^*^ + *t*(*y* ^*^ − *x* ^*^))*dt* exists and

(33) $$\begin{array}{c} \overset{1}{\underset{0}{\int }}F^{\prime }\left(x^{*}+t\left(y^{*}-x^{*}\right)\right)dt\left(y^{*}-x^{*}\right)=F\left(y^{*}\right)-F\left(x^{*}\right)=0. \end{array}$$

We get that *x* ^*^ = *y* ^*^.

This completes the proof of Theorem 6.

## 3. Application

In this section, we apply the convergence theorem and show three numerical examples.

**Example 1 .**

Consider the root of the equation *F*(*x*) = *x* ^10/3^ + *x* ^7/2^ − *x* − 1 = 0 on *x* ∈ (0, +*∞*). Then, we easily get that

(34) $$\begin{array}{c} F^{\prime \prime \prime }\left(x\right)=\frac{280}{27}x^{1/3}+\frac{105}{8}x^{1/2} \end{array}$$

does not satisfy (*K*, *p*) Hölder condition

(35) $$\begin{array}{c} \left\|F^{\prime \prime \prime }\left(x\right)-F^{\prime \prime \prime }\left(y\right)\right\|\leq K{\left\|x-y\right\|}^{p} \end{array}$$

because, for all *p* ∈ (0,1],

(36) $$\begin{array}{c} \underset{x,y\in (0,+\infty )}{\sup}\frac{\left(280/27\right){\left|x-y\right|}^{1/3}+\left(105/8\right){\left|x-y\right|}^{1/2}}{{\left|x-y\right|}^{p}}=+\infty . \end{array}$$

Let

(37) $$\begin{array}{c} \omega \left(z\right)=\frac{280}{27}z^{1/3}+\frac{105}{8}z^{1/2},\;z>0; \end{array}$$

then *ω*(*tz*) ≤ *t* ^1/3^ *ω*(*z*) for *t* ∈ [0,1] and *z* ∈ [0, +*∞*);

(38) $$\begin{array}{c} \left\|F^{\prime \prime \prime }\left(x\right)-F^{\prime \prime \prime }\left(y\right)\right\|\leq \omega \left\|x-y\right\|. \end{array}$$

Let us consider a particular case of 2 from the operator given by the following nonlinear integral equation of mixed Hammerstein type (see [26]):

(39) $$\begin{array}{c} x\left(s\right)=\alpha \left(s\right)-\overset{m}{\underset{i=1}{\sum }}\overset{b}{\underset{a}{\int }}k\left(s,t\right)\varphi_{i}\left(x\left(t\right)\right)dt, \end{array}$$

where −*∞* < *a* < *b* < +*∞*, *u*, *φ*_*i*_, for *i* = 1,2,…, *m*, are known functions and *x* is a solution to be determined. If *φ*′′′ is (*L*_*i*_, *p*_*i*_) Hölder continuous in *Ω*, for *i* = 1,2,…, *m*, the corresponding operator *F* : *Ω*⊆*C*[*a*, *b*] → *C*[*a*, *b*],

(40) Fxs=xs+∑i=1m∫abks,tφixtdt−αs,s∈a,b,

does not satisfy (*K*, *p*) Hölder condition; for instance, the max-norm is considered. In this case,

(41) F′′′(x)−F′′′(y)≤∑i=1mLix−ypi,Li>0, pi∈0,1, x,y∈Ω.

To solve this type of equations, we can consider

(42) $$\begin{array}{c} \left\|F^{\prime \prime \prime }\left(x\right)-F^{\prime \prime \prime }\left(y\right)\right\|\leq \omega \left(\left\|x-y\right\|\right),\;x,y\in \Omega , \end{array}$$

where *ω*(*z*) = Σ_*i*=1_^*m*^ *L*_*i*_ *z* ^*p*_*i*_^ satisfy *ω*(*tz*) ≤ *t* ^*q*^ *ω*(*z*), where *q* = min⁡{*p*_*i*_, *p*_2_,…, *p*_*m*_}.

**Remark 7 .**

Observe that if *F*′′′ is Lipschitz continuous in *Ω*, we can choose *ω*(*z*) = *Kz*, *K* > 0, so that Jarratt's method is of *R*-order, at least four order. If *F*′′′ is (*L*, *p*) Hölder continuous in *Ω*, then we can choose *ω*(*z*) = *Lz* ^*p*^, *L* < 0, *p* ∈ (0,1], and Jarratt's method is of *R*-order, at least 3 + *p*.

**Example 2 .**

Consider the case as follows:

(43) xs=1+132∫01k(s,t)xt16/5dt +130∫01k(s,t)xt10/3dt,

where the space is *X* = *C*[0,1] with the norm

(44) $$\begin{array}{c} \left\|x\right\|=\underset{0\leq s\leq 1}{\max}\left|x\left(s\right)\right|,\\ k\left(s,t\right)=\left\{\begin{array}{cc} t\left(1-s\right), & t\leq s,\\ s\left(1-t\right), & s\leq t. \end{array}\right. \end{array}$$

This equation arises in the theory of the radiative transfer and neutron transport and in the kinetic theory of gasses. Let us define the operator *F* on *X* by

(45) Fx=x(s)−132∫01k(s,t)xt16/5dt −130∫01k(s,t)xt10/3dt−1.

The first, the second, and the third derivatives of *F* are defined by

(46) F′xus=u(s)−110∫01k(s,t)xt11/5u(t)dt −19∫01k(s,t)xt7/3u(t)dt, u∈X,F′′xuvs=−1150∫01k(s,t)xt6/5u(t)v(t)dt −727∫01k(s,t)xt43u(t)v(t)dt,            u∈X,F′′′xuvws=−66250∫01k(s,t)xt1/5u(t)v(t)w(t)dt −2881∫01k(s,t)xt1/3u(t)v(t)w(t)dt,

and we have

(47) [F′′′(x)−F′′′(y)]uvw ≤66250max⁡s∈[0,1]  ×∫01k(s,t)xt1/5−yt1/5u(t)v(t)w(t)dt  +2881max⁡s∈[0,1]  ×∫01k(s,t)xt1/3−yt1/3u(t)v(t)w(t)dt ≤66250×18x−y1/5uvw  +2881×18x−y1/3uvw.

To apply Theorem 6, we choose *x*_0_ = *x*_0_(*s*) = 1 and we look for a domain in the form

(48) $$\begin{array}{c} \Omega =B\left(1,2\right)\subseteq C\left(\left[0,1\right]\right). \end{array}$$

In this case, we have

(49) $$\begin{array}{c} \left\|I-F^{\prime }\left(x_{0}\right)\right\|\leq \frac{19}{720}<1 \end{array}$$

and from the Banach lemma, we obtain

(50) F′x0−1≤720701,F′x0−1F(x0)≤720701×18132+130=η=9311216,M=0.148766⋯,  N=0.0948511⋯,ω(z)=33100z1/5+7162z1/3,  p=15.

Then *a*_0_ = *Mη* = 0.00123353 < 1/2, *b*_0_ = 6.52127 × 10^−6^, *c*_0_ = 1.47132 × 10^−6^, *γ* = *f* ^2^(*a*_0_)*g*(*a*_0_, *b*_0_, *c*_0_) = 3.48167 × 10^−7^ < 1, Δ = 0.998766⋯, and *R* = 1.00062⋯. This means that the hypothesis of Theorem 6 is satisfied. Then, the error bound becomes

(51) x∗−xn ≤1+γ3.2n−1/2.2a021+γ3.2n−1/2.2a0  ×11−γ3.2nΔγ(3.2n−1/2.2)Δnη.

For *n* = 1,2, 3,4, we get

(52) $$\begin{array}{c} \left\|x_{1}-x^{*}\right\|\leq 4.28944\times 10^{-10},\\ \left\|x_{2}-x^{*}\right\|\leq 5.76451\times 10^{-16},\\ \left\|x_{3}-x^{*}\right\|\leq 6.63209\times 10^{-23},\\ \left\|x_{4}-x^{*}\right\|\leq 2.86064\times 10^{-32}. \end{array}$$

**Example 3 .**

Let us consider the system of equations *F*(*u*, *v*) = 0, where

(53) $$\begin{array}{c} F\left(u,v\right)={\left(u^{7/2}-uv-v^{10/3}+1,u^{7/2}+uv-v^{10/3}-1\right)}^{T}. \end{array}$$

Then, we have

(54) F′(u,v)=72u5/2−v−103v7/3−u72u5/2+v−103v7/3+u,F′u,v−1=114/2u7/2+20/3v10/3 ×−103v73+u103v73+u−72u52−v72u52−v,,F′′(u,v)=354u3/2−1−1−709v4/3354u3/211−709v4/3.,F′′′(u,v)s,t3=1058u1/228027v1/31058u1/228027v1/3s3t3.

Now, we choose *x*_0_ = (*u*_0_, *v*_0_) = (1.5,1.5) and *Ω* = {*x*∣‖*x* − *x*_0_‖ ≤ 1.5}. We take the max-norm in *R* ^2^ and the norm ‖*A*‖ = max{|*a*_11_| + |*a*_12_|, |*a*_21_| + |*a*_22_|} for $A=\left(\begin{array}{cc} a_{11} & a_{12}\\ a_{21} & a_{22} \end{array}\right)$. We define the norm of a bilinear operator *B* on *R* ^2^ by

(55) $$\begin{array}{c} \left\|B\right\|=\underset{\left\|u\right\|=1}{\sup}\underset{i}{\max}\overset{2}{\underset{j=1}{\sum }}\left|\overset{2}{\underset{k=1}{\sum }}b_{i}^{jk}u_{k}\right|, \end{array}$$

where *u* = (*u*_1_, *u*_2_)^*T*^ and $B=\left(\begin{array}{cc} b_{1}^{11} & b_{1}^{12}\\ b_{1}^{21} & b_{1}^{22}\\ b_{2}^{11} & b_{2}^{12}\\ b_{2}^{21} & b_{2}^{22} \end{array}\right)$.

Then, we get the following results: *η* = ‖*F*′(*x*_0_)^−1^ *F*(*x*_0_)‖ = 0.09598⋯, *M* = 9.20456⋯, *N* = 10.7635⋯, and *p* = 1/3.

We get that the hypotheses of Theorem 6 are satisfied.
